# Supplementary material for: Insight into the Inhibitory Mechanism of Aryl Formyl Piperidine Derivatives on Monoacylglycerol Lipase through Molecular Dynamics Simulations
Source: Molecules. 2022 Nov 3;27(21):7512. doi: 10.3390/molecules27217512 (PMC9655140; doi:10.3390/molecules27217512)
Supplement: Supplementary file 1 [file molecules-27-07512-s001.zip › molecules-2004469-supplementary.pdf]

Insight into the inhibitory mechanism of Aryl Formyl Piperidine derivatives on Monoacylglycerol Lipase through Molecular Dynamics Simulations

Chang Liu [1], Shanshan Guan [2,3], Jingwen E [1], Zhijie Yang [1], Xinyue Zhang [1,] Jianan Ju [1], Song Wang [1,*]and Hao Zhang [1,*]

[1] Laboratory of Theoretical and Computational Chemistry, Institute of Theoretical Chemistry, College of Chemistry, Jilin University, Changchun 130023, China

[2] College of Biology and Food Engineering, Jilin Engineering Normal University, Changchun 130052, China

[3] Key Laboratory of Molecular Nutrition at Universities of Jilin Province, Changchun 130052, China

* Correspondence: ws@jlu.edu.cn (S.W.); stringbell@jlu.edu.cn (H.Z.); Tel.: +86-0431-88498761 (S.W. & H.Z.)

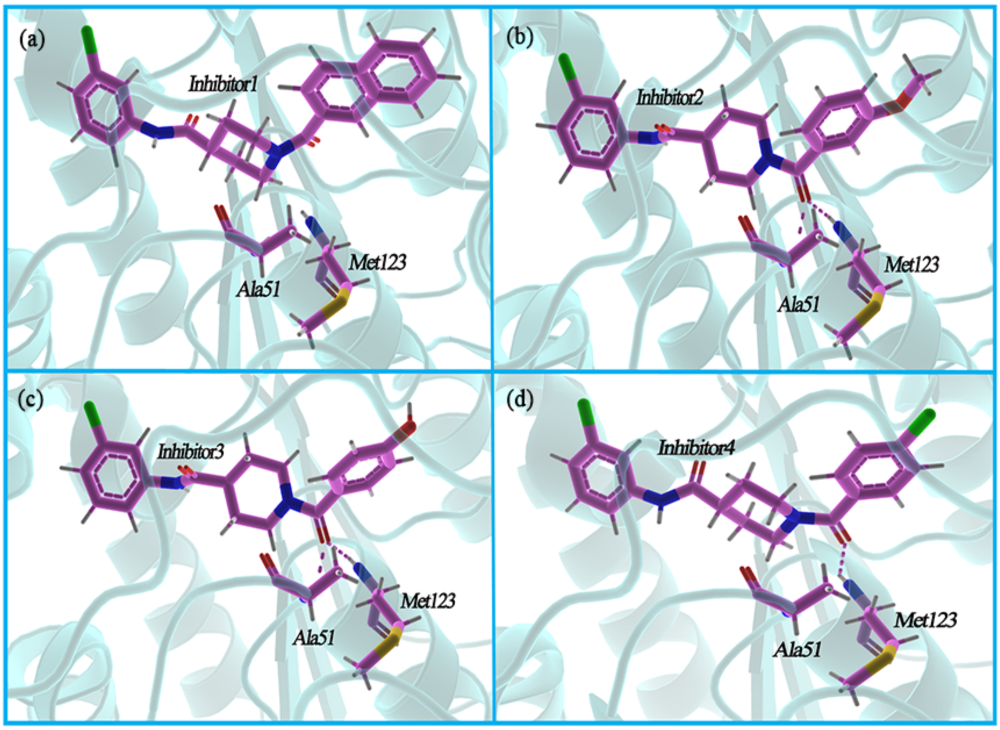

Figure S1. A diagram of the initial conformational patterns of the four complexes obtained by docking,(a)Complex 1, (b)Complex 2, (c)Complex 3, (d)Complex 4.

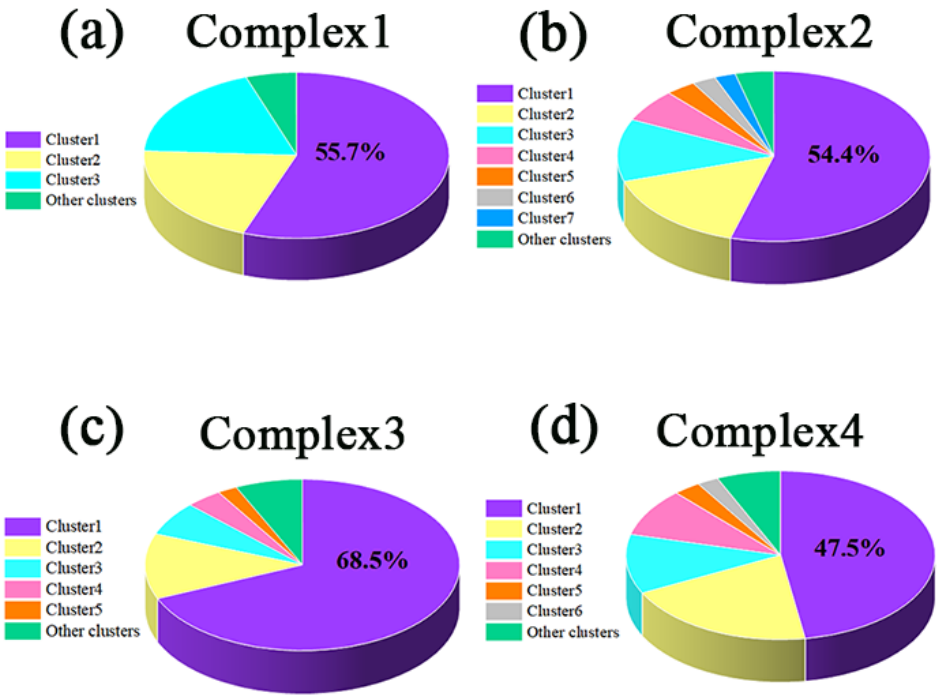

Figure S2. The results of the RMSD-based clustering of the four complexes are shown in the figure, with each complex obtaining its corresponding representative clusters and the "other clusters" being representative clusters that have less than 2% of the capacity.

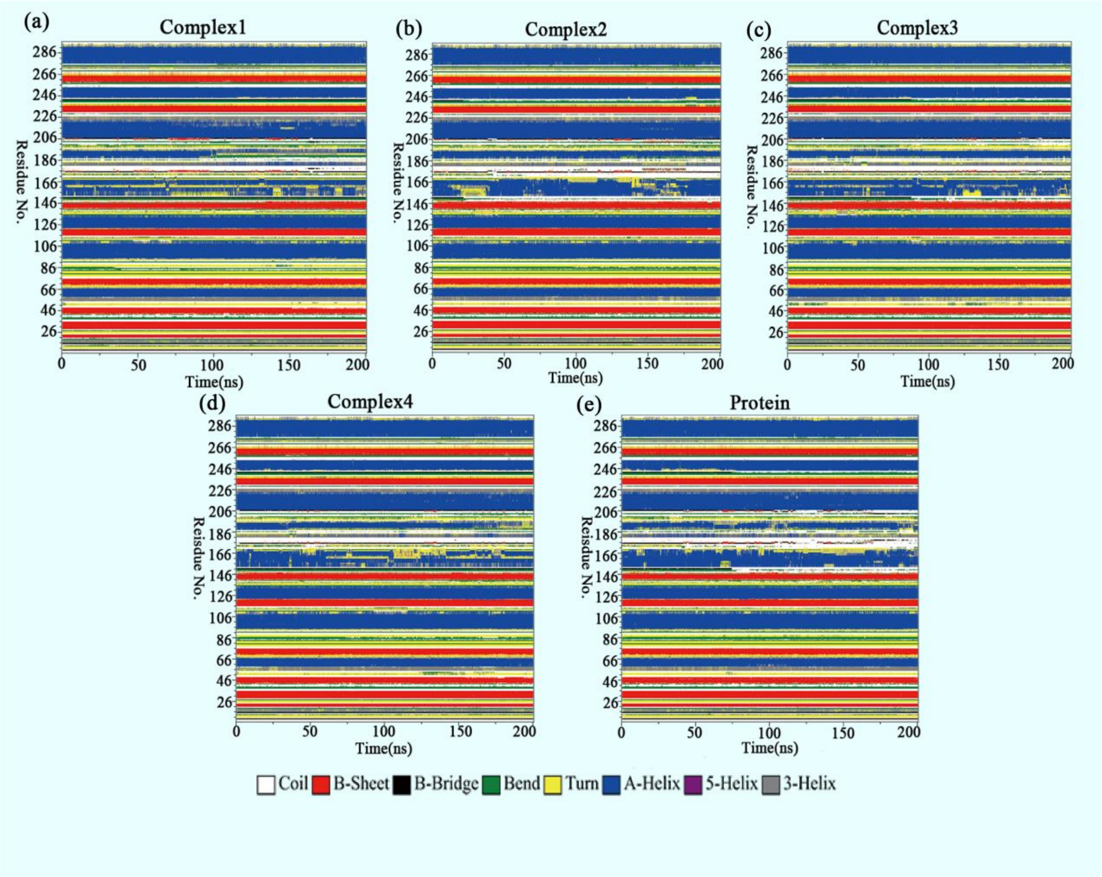

Figure S3. The plot of the whole secondary structure species of the four complexes and the protein with simulation time.

Table S1. The percentages of the first five eigenvectors of the four complexes.

Systems	PC1	PC2	PC3	PC4	PC5	Total
Complex1	25.2%	11.1%	7.0%	4.3%	10.3%	57.9%
Complex2	22.9%	12.8%	7.3%	4.1%	10.9%	58.0%
Complex3	16.8%	13.4%	7.4%	4.6%	12.6%	54.8%
Complex4	22.5%	13.3%	8.2%	5.2%	11.0%	60.2%

Table S2. The various decomposition energies of the important residues in the four complexes (kJ·mol^{-1}).

Residues	Complex1	Complex2	Complex3	Complex4
Gly50	-4.5096[a]	-9.6398	-9.9206	-2.3282
	3.9624[b]	4.1893	4.6423	1.3702
	-0.0540[c]	-0.0389	-0.0563	-0.0095
	-0.6027[d]	-5.4894	-5.3370	-0.9684
Ala51	-14.2399	-16.0677	-16.6692	-5.8165
	13.0334	11.5887	11.8617	7.6964
	-0.5967	-0.5691	-0.7097	-0.3685
	-1.8033	-5.0454	-5.5175	1.5163
Glu53	-32.3265	-34.2925	-21.4421	4.0425
	41.2708	42.1167	35.9210	3.8122
	-0.5069	-0.4377	-0.3528	-0.0755
	8.4268	7.3743	14.1181	7.7922
Ser122	-18.9881	-11.1333	-11.9032	-3.8783
	11.3517	7.7205	7.0439	4.9793
	-0.2698	-0.4522	-0.4570	-0.1786
	-7.9111	-3.8577	-5.3147	0.9229
Met123	-12.1313	-12.1920	-14.5132	-2.5306
	4.5824	4.7105	4.9351	2.0820
	-0.0435	-0.1036	-0.1205	-0.0425
	-7.5908	-7.5904	-9.7019	-0.4924
Ser155	-4.7892	0.4058	-0.2051	-4.4302
	6.7868	-0.2715	0.4622	3.6556
	-0.2302	-0.0339	-0.0647	-0.1329
	1.7672	0.1004	0.1920	-0.9096
Ile179	-1.7921	-1.2348	-2.1505	-3.5604
	0.3510	0.0132	-0.1957	1.4116
	-0.0209	-0.2416	-0.5795	-0.3671
	-1.4618	-1.4659	-2.9276	-2.5138
Ser181	-1.0483	-2.9446	-6.8193	-4.2465
	1.2266	3.4855	3.8107	3.2641
	-0.0692	-0.1588	-0.1808	-0.1717
	0.1074	0.3838	-3.1917	-1.1514
Leu184	-6.6556	-4.9022	-6.3498	-2.7562
	0.4401	0.7352	0.8384	-0.2558
	-0.3767	-0.3838	-0.4904	-0.3527
	-6.5920	-4.5502	-6.0014	-3.3643
Glu190	-5.1072	-1.6855	0.6049	1.6572
	4.6991	3.1748	2.5685	1.1958
	-0.1103	-0.0657	-0.0988	-0.0642

	**-0.5189**	**1.4289**	**3.0747**	**2.7899**
**Val191**	**-2.5694**	**-2.0690**	**-2.0039**	**-1.9634**
	**0.9545**	**0.8746**	**0.6006**	**0.5774**
	**-0.0904**	**-0.0994**	**-0.1061**	**-0.1592**
	**-1.7028**	**-1.2950**	**-1.5110**	**-1.5430**
**Tyr194**	**-12.0189**	**-14.0364**	**-17.6955**	**-7.3541**
	**6.2679**	**8.2303**	**9.6291**	**4.9009**
	**-0.8757**	**-1.0501**	**-1.1365**	**-0.9373**
	**-6.6294**	**-6.8613**	**-9.2035**	**-3.3842**
**Asp197**	**-5.0952**	**-0.6685**	**1.2170**	**-0.6643**
	**3.2962**	**1.1557**	**0.4084**	**0.9553**
	**0.0002**	**0.0006**	**0.0000**	**-0.0008**
	**-1.7977**	**0.4862**	**1.6243**	**0.2916**
**Leu205**	**-1.3405**	**-3.7223**	**-1.8572**	**-4.4476**
	**-0.1636**	**-0.0461**	**-0.2341**	**0.3072**
	**-0.3953**	**-0.5987**	**-0.4126**	**-0.4852**
	**-1.9001**	**-4.3660**	**-2.5040**	**-4.6279**
**Leu213**	**-4.9730**	**-2.7714**	**-3.6157**	**-2.4997**
	**0.2927**	**0.1268**	**0.2246**	**0.2716**
	**-0.4793**	**-0.3252**	**-0.4528**	**-0.2283**
	**-5.1605**	**-2.9704**	**-3.8434**	**-2.4593**
**Asp239**	**6.1861**	**-0.0979**	**-4.4447**	**2.4919**
	**-5.8245**	**-2.3503**	**-2.5186**	**-3.0454**
	**0.0004**	**0.0004**	**0.0000**	**0.0001**
	**0.3575**	**-2.4502**	**-6.9640**	**-0.5526**
**Leu241**	**-11.1951**	**-8.5101**	**-5.2921**	**-7.0123**
	**4.4685**	**4.3976**	**1.0552**	**2.6352**
	**-0.8278**	**-1.1393**	**-0.8066**	**-0.6989**
	**-7.5579**	**-5.2549**	**-5.0399**	**-5.0822**
**His269**	**-25.4018**	**-3.1805**	**7.0066**	**-11.3696**
	**26.7227**	**14.4599**	**16.4347**	**14.0516**
	**-0.1303**	**-0.0409**	**-0.0975**	**-0.0323**
	**1.2032**	**11.2382**	**23.3346**	**2.6477**
**Val270**	**-5.5963**	**-6.0451**	**-4.7169**	**-4.5508**
	**0.3910**	**0.4089**	**0.1763**	**0.5846**
	**-0.5290**	**-0.6817**	**-0.7396**	**-0.4795**
	**-5.7358**	**-6.3203**	**-5.2817**	**-4.4466**
**Lys273**	**0.8941**	**-0.2677**	**-3.7450**	**-7.3273**
	**2.7429**	**1.1238**	**3.3418**	**5.5281**
	**-0.2031**	**-0.3149**	**-0.1260**	**-0.1850**
	**3.4319**	**0.5412**	**-0.5295**	**-1.9815**

(a:ΔE_{MM} b:ΔG_{polar} c:$\Delta G_{nonpolar}$ d:$\Delta G_{binding}$)
